# Ca^2+^/Calmodulin-Dependent Protein Kinase II Contributes to Hypoxic Ischemic Cell Death in Neonatal Hippocampal Slice Cultures

**DOI:** 10.1371/journal.pone.0070750

**Published:** 2013-08-19

**Authors:** Qing Lu, Valerie A. Harris, Xutong Sun, Yali Hou, Stephen M. Black

**Affiliations:** Vascular Biology Center, Georgia Regents University, Augusta, Georgia, United States of America; Albany Medical College, United States of America

## Abstract

We have recently shown that p38MAP kinase (p38MAPK) stimulates ROS generation via the activation of NADPH oxidase during neonatal hypoxia-ischemia (HI) brain injury. However, how p38MAPK is activated during HI remains unresolved and was the focus of this study. Ca^2+^/calmodulin-dependent protein kinase II (CaMKII) plays a key role in brain synapse development, neural transduction and synaptic plasticity. Here we show that CaMKII activity is stimulated in rat hippocampal slice culture exposed to oxygen glucose deprivation (OGD) to mimic the condition of HI. Further, the elevation of CaMKII activity, correlated with enhanced p38MAPK activity, increased superoxide generation from NADPH oxidase as well as necrotic and apoptotic cell death. All of these events were prevented when CaMKII activity was inhibited with KN93. In a neonatal rat model of HI, KN93 also reduced brain injury. Our results suggest that CaMKII activation contributes to the oxidative stress associated with neural cell death after HI.

## Introduction

Neonatal hypoxia ischemia (HI) brain injury occurs in ∼2–5/1000 births. Approximately 30–40% of infants with brain injury will die and 20–40% of survivors develop significant neurological disorders and lifelong disability, including cerebral palsy, seizures, visual impairment, mental retardation, learning impairment and epilepsy [1], [2], [3]. The main mechanisms underlying neurological damage in HI are oxygen and glucose deprivation, which leads to energy failure, following a cascade of biochemical events such as Ca^2+^ influx, increased permeability of cell membranes and oxidative stress. The consequent reperfusion often exacerbates the injury by increasing the oxidative damage. It is well established that energy failure, increases in intracellular Ca^2+^ and overproduction of reactive oxygen species (ROS) play major roles in cell death for both immature and mature brains after HI [4], [5], [6]. The immature brain may be more vulnerable to oxidative damage than adult due to high concentration of unsaturated fatty acids, high rate of oxygen consumption, and availability of redox-active iron [7], [8], [9], [10], [11]. There are several systems responsible for the increase in ROS associated with neonatal HI including uncoupled NOS [12], the mitochondria [13], [14] and potentially xanthine oxidase [15]. In addition, we have recently shown that p38MAP kinase (p38MAPK) stimulates ROS generation via the activation of NADPH oxidase during neonatal HI injury [16]. However, it is unresolved how p38MAPK is activated during neonatal HI [16], [17].

CaMKII is involved in the regulation of synaptogenesis and plasticity during development [18], [19], [20]. Neural Ca^2+^ binds to calmodulin (CaM) forming a Ca^2+/^CaM complex, which in turn activates CaMKII through its autophosphorylation at Thr286, Thr305, and Thr306. It has been reported that CaM antagonists can inhibit cell death and ischemic brain damage [21], [22], [23]. Interestingly, inhibition of CaMKII has also been shown to be neuroprotective [24], [25]; however, the underlying mechanism remains to be elucidated. In addition, we have recently shown that the activation of NADPH oxidase during neonatal HI is mediated by the phosphorylation of p47^phox^ by p38MAPK. In this study we investigated if CaMKII is the upstream regulator of p38MAPK and if so whether CaMKII inhibition can attenuate the neural cell death associated with neonatal HI.

## Methods

### Hippocampal Slice Culture and OGD Exposure

Neonatal rats (Sprague-Dawley, Charles River, Wilmington, MA, USA) at postnatal Day 7 (P7) were decapitated and the hippocampi dissected under sterile conditions. Each hippocampus was sliced into 400 µm slices using a Mcllwain tissue chopper (Science Products GmbH, Switzerland). Slices were then cultured on permeable membrane Millicell inserts (Millipore, Billerica, MA, USA) (0.4 µm pore size) in six well plates for 6 days at 37°C in 5% CO_2_ as previously described [16], [17]. Twenty-four hours before exposure to OGD the culture medium was changed to neurobasal-A and B27 supplement minus antioxidants. Just prior to OGD, a sucrose balanced salt solution (SBSS) (120 mM NaCl, 5 mM KCl, 1.25 mM NaH_2_PO_4_, 2 mM MgSO_4_, 2 mM CaCl_2_, 25 mM NaHCO_3_, 20 mM HEPES, 25 mM sucrose, pH of 7.3) was infused for 1 hour with 5%CO_2_ and 10 L/h nitrogen gas. The inserts were then transferred into deoxygenated SBSS and placed in a ProOxC system chamber with oxygen controller (BioSpherix, NY, USA) and exposed to 0.1% O_2_, 5%CO_2_, 94.4% nitrogen for 90 min at 37°C. The slices were then returned to oxygenated serum-free neurobasal medium with B27 supplement. The CaMKII inhibitor, KN93 *N*-[2-[[[3-(4-Chlorophenyl)-2-propen­yl]methylamino]methyl]phenyl]-*N*-(2-hydroxyethyl)-4–methoxybenzenesulphonamide (EMD Biochemicals, San Diego, CA, USA) or the inactive analog KN92 2- [N- (4- Methoxybenzenesulfonyl)]amino- N- (4- chlorocinnamyl)- N- methylbenzylamine, monohydrochloride (EMD Biochemicals, San Diego, CA, USA) were dissolved in dimethyl sulphoxide (DMSO) and added to the medium 2 h before OGD. Another CaMKII inhibitor, autocamitide 2-related inhibitor peptide (AIP) with the sequence Lys-Lys-Ala-Leu-Arg-Arg-Gln-Glu-Ala-Val-Asp-Ala-Leu (#A4308-1MG, Sigma, St. Louis, MO) was also utilized. In these experiments, AIP was added to the medium 2 h before OGD. Control experiments contained the equivalent amount of DMSO that did not exceed 0.2% (v/v). All protocols were approved by the Institutional Animal Care and Committee at Georgia Regents University.

### CaMKII Activity

CaMKII activity was measured using the CycLex Calmodulin kinase II Assay Kit (CycLex Co., Nagano, Japan). Briefly, hippocampal slice cultures or isolated rat brain hippocampii were washed with ice-cold PBS and lysed in lysis buffer containing 1% Triton X-100, 20 mM Tris, pH 7.4, 100 mM NaCl, with 1× protease inhibitor cocktail, and 1× phosphatase inhibitor cocktail (Sigma, St. Louis, MO, USA). Samples were sonicated on crushed ice with two 10 s bursts and centrifuged at 13,000 g for 5 min at 4°C. Supernatants were treated according to the manufacturer's instructions and the protein content was measured. Samples were diluted 1∶10 in the kinase buffer provided with the kit, containing 20 mM ATP, and incubated for 60 min at 30°C in 96-well plates, pre-coated with a synthetic target polypeptide for CaMKII. Wells were washed 5 times with 2% (v/v) Tween-20. Then 100 µl of the horseradish peroxidase-conjugated anti-phospho Syntide-2 monoclonal antibody was added. After 60 min incubation at room temperature, samples were washed again, and the chromogenic substrate, tetramethylbenzidine (100 µl) was added. After a 15 min incubation at room temperature, the reaction was stopped by the addition of 0.5 N H_2_SO_4_ (100 µl) and the absorbance read at 450 nm, using a microplate reader (Synergy HT, BioTek Instruments, Vermont, USA) [26], [27]. It should be noted that syntide-2, is not totally specific for CaMKII. However, the relative V_max_/K_m_ ratios of the known Ca^2+^-dependent protein kinases for syntide-2 are as follows: CaMKII, 100; protein kinase C, 22; phosphorylase kinase, 2; myosin light chain kinase, 0.005 [28]. Thus, this assay is highly preferential for estimating CaMKII activity.

### Propidium Iodide (PI) Staining

Quantification of slice culture cell death was carried out 8 h after OGD using PI staining and fluorescent microscopy. This time point was chosen based on our prior published studies [16], [17]. Briefly, PI (1 µg/mL; Sigma) was added to the culture medium 24 h prior to OGD. Slice cultures were then examined prior to OGD with an inverted fluorescence microscope (Olympus IX51; Japan) using an excitation wavelength of 510 nm and an emission wavelength of 590 nm. Slices showing distinct PI intake were excluded from further study. Images were then obtained at baseline and 8 h after OGD using a 10-bit monochrome fluorescence camera (Digital Camera C4742-95; Hamamatsu, Japan. Images were then processed using Image-Pro Plus 6.0 (Media Cybernetics, Silver Spring, MD, USA). The evaluation of cell death was then determined using a modification of the method of Cronberg et al [29] as we have recently described [16], [17].

### LDH Cytotoxicity Assay

Cytotoxicity was evaluated by quantification of lactate dehydrogenase (LDH) using a Cytotoxicity Detection Kit (Roche Applied Science, Mannheim, Germany) in the slice culture medium as described [16], [17]. Samples were analyzed 8 h after OGD. All LDH measurements were normalized using total protein levels (Bradford protein assay, Bio-Rad Laboratories, CA, USA).

### Histologic Evaluations

Slice cultures were washed in PBS, fixed in 4% paraformaldehyde (RT, 1 h), then in 30% sucrose (RT, 1 h), embedded in O.C.T embedding medium (Tissue-Tek, Sakura Fine technical, Tokyo, Japan) and stored at −80°C overnight. Embedded tissues were sectioned (15 µm), mounted on glass slides and stored at −80°C until used. In the rat HI brain model, pups were anesthetized with 75 mg Ketamine/10 mg Xylazine cocktail i.p., followed by transcardial saline extravasation and perfused with 4% paraformaldehyde in 0.1M phosphate buffer, pH 7.4. The brains were post-fixed for 24 h at 4°C and cryoprotected in 30% sucrose before sectioning. Permeabilized sections were analyzed for the presence of apoptotic nuclei using the DeadEnd Fluorometric TUNEL System (Promega, Madison, WI, USA) as described [16], [17]. Quantification of the TUNEL stained nuclei and total nuclei was processed by Image-Pro software and presented as a percentage of total nuclei in the field using PI to label the nuclei.

### Immunoblot Analyses

Slice cultures were washed with ice-cold phosphate-buffered saline, homogenized in lysis buffer containing 1% Triton X-100, 20 mM Tris, pH 7.4, 100 mM NaCl, with 1× protease inhibitor cocktail, and 1× phosphatase inhibitor cocktail (Sigma, St. Louis, MO, USA). Lysates were centrifuged at 13,000 *g* for 10 min at 4°C to precipitate the debris, and the protein content in the supernatant determined using the Bio-Rad protein assay (Bio-Rad Laboratories, CA USA). Lysate protein (20 µg/lane) was separated using 4–20% gradient gels (Thermo Scientific, Rockford, IL, USA) and transferred to PVDF membranes. The blots were then probed with the appropriate antibody overnight at 4°C. Primary antibodies used were anti-p47^phox^ (Cell Signaling, Danvers, MA, USA); anti-p38MAPK (#9212S, Cell Signaling, Danvers, MA, USA); anti-phospho-p38MAPK (#4631S, Cell Signaling, Danvers, MA, USA); anti Na^+^K^+^ ATPase (a plasma membrane marker, *ab8344*, Abcam Inc., Cambridge, MA, USA); anti-VDAC (a mitochondrial membrane marker, ab34726, Abcam, Inc., Cambridge, MA, USA); anti-Lamin B1 (a nuclear membrane marker, ab8982, Abcam Inc., Cambridge, MA, USA), and anti-cleaved caspase 3 (Cell signaling, Danvers, MA, USA). Blots were washed in 1 X TBST (3×15 min) and the appropriate secondary antibodies conjugated to HRP were then added for 1 h at RT (Thermo Scientific, Rockford, IL, USA). After further washing in TBST (3×15 min) bands were visualized by chemiluminescence (West-Femto, Pierce, Rockford, IL, USA) and quantified using a Kodak Molecular Imaging System (Kodak, Rochester, NY, USA). The plasma membrane was isolated using the Pierce Mem-PER Eukaryotic Membrane Protein Extraction Reagent Kit according to manufacturer's protocol (Thermo Fisher Scientific, Rockford, IL, USA).

### Measurement of Superoxide Levels

Superoxide production was measured using electron paramagnetic resonance (EPR) spectroscopy as we have previously described [16], [17]. To quantify the amount of superoxide generated, waveform amplitudes were normalized to those generated by xanthine-xanthine oxidase. Under standard reaction conditions, 1 unit of xanthine oxidase converts 1 µmol of xanthine per minute at 25°C. From the resulting standard based curve, a coefficient of 303.6 EPR amplitude units/µmol of superoxide was produced in our reaction. The waveform amplitudes generated in slice cultures of brain hippocampi were converted into nanomoles of superoxide per milligram/minute of protein utilizing this value.

### NADPH Oxidase Activity

Hippocampal tissue was collected and homogenized with Tris-sucrose buffer (10 mM Tris base (Fisher, Pittsburgh, PA, USA), 340 mM sucrose (Mallinkrodt Baker, Inc Philipsburg, NJ, USA), 1 mM EDTA (Mallinkrodt Baker, Inc Philipsburg, NJ, USA), 10 ug/ml protease inhibitor mixture (Sigma, St. Louis, MO, USA)). The homogenate protein concentration was measured. NADPH oxidase activity was measured by a luminescence assay in the reaction buffer with 5 µM Lucigenin, 1 mM EGTA and 50 mM phosphate buffer, pH 7.0. One hundred micrograms of homogenate protein +100 µM NADPH as substrate (Sigma, St. Louis, MO, USA) were added to 8 mm test tubes containing 500 µl of reaction buffer, then incubated at 37°C for 5 min. Photon emission was measured for 15 sec in a luminometer (model TD-20/20, Turner Designs, Sunnyvale, CA, USA).

### Rat Model of Neonatal Hypoxia-Ischemia

The modified Levine procedure was conducted on Sprague-Dawley postnatal day 7 (P7) [30]. Briefly, rat pups were anesthetized with isoflurane (4% for induction; 3% for maintenance), and 20% oxygen at 1 L/min flow rate. For the duration of induction and surgery, a Microflex EZ Anesthesia System equipped with a heated induction chamber and surgical bed (Euthanex, Palmer, PA, USA) maintained core body temperature between 35–36°C. Rectal temperature was monitored continuously using a sensitive microprobe thermometer (BAT-12, Physitemp Instruments, Clifton, NJ, USA). After a midline incision to the neck, the right common carotid artery was located, separated from the vagus nerve, and permanently occluded approximately 3–4 mm distal to the ECA-CCA bifurcation by electrical coagulation (KMLS, Martin ME102; Harvard Apparatus, Holliston, MA). The incision was then closed with 6-0 silk suture. Sham operated pups received vessel manipulation without occlusion. All vessel occlusions were completed during a 5 minutes period of anesthetic maintenance. Immediately after surgery, pups were placed in a temperature controlled recovery chamber for 15 min before being returned to the dam for 1–2 h. Thirty minutes before the end of dam recovery, the pups were reanesthetized with isoflurane, administered an intracerebroventricular injection of KN93, KN92 or vehicle into the right hemisphere, as described above, then returned to the heated chamber. To induce HI, pups were placed in a custom made Plexiglass hypoxia device (Jarrold Manufacturing, St. Louis, MO, USA) [31], [32], [33]. A heated immersion circulator (Isotemp, Fisher Scientific, USA) was used to maintain the temperature of six interconnected chambers and one adjacent chamber (offset and unconnected from the other six) submerged in a water bath at 36.6°C to maintain chamber ambient and floor temperatures of 34–34.5°C and 35.5°C, respectively. Each chamber, housing 1–2 pups, was infused with a commercially calibrated mixture of warm, humidified 8% oxygen/balance nitrogen for 2.5 hours at a flow rate of 100 ml^3^/min. The oxygen concentration was monitored using a Mini-Ox 3000 oxygen analyzer (MSA Medical Products, Pittsburgh, PA, USA) and core body temperature of pups maintained between 35–36°C. Immediately after hypoxia, pups were removed from the chamber and placed in recovery chamber (36°C), and allowed 1–2 hours of recovery before being returned to the dam. At 2- and 24-h post-ischemic recovery, the brains of the pups were dissected for further analysis. KN93 (EMD Biochemicals, San Diego, CA, USA) was supplied as a water-soluble powder, diluted to working stock concentration of 5 mM in saline. KN92 (EMD Biochemicals, San Diego, CA, USA) was supplied as solution of DMSO, diluted to 5 mM in DMSO. Working stocks were diluted to their final concentrations in artificial cerebrospinal fluid containing 0.2% Trypan Blue and 2% DMSO. Trypan blue was used as tracer dye to ascertain location of stereotactic coordinates, and as an indicator of injection accuracy. Thirty minutes before the end of postsurgical dam recovery, and before the onset of HI, a 3 µl injection volume containing 0.3 or 0.6 nmol KN93 or KN92, was delivered to the lateral ventricles of the right hemisphere in similar fashion to previously published [16]. For the vehicle treatments, artificial cerebrospinal fluid containing 0.2% Trypan Blue and 2% DMSO was similarly injected. The initial drug dose of 0.1 mM and injection volume was chosen from previous published procedures for CaMKII inhibitor i.c.v administration [34]. Also included was a sham operated group (n = 6), that received carotid isolation with manipulation only and no exposure to hypoxia. The Levine groups (vessel occlusion with HI) were divided into five subgroups, (n = 6 per group). An additional group (n = 6) received the higher ventricular dose of KN93 (0.6 nmol), in the absence of hypoxia.

### Analysis of Brain Infarct Size

After swift removal and dissection, brains were placed in chilled 0.15M PBS for 5 min before using a rodent neonatal matrix (Zivic Instruments, Pittsburgh, PA, USA) to make five 2 mm thick coronal sections. Sections from rostra tip to caudal end of forebrain, were immersed in 2% TTC (2,3,5-triphenyl-tetrazolium) (Sigma, St. Louis, MO, USA) dissolved in 0.15M PBS, incubated for 30 min at room temperature followed by two rinses in chilled PBS on ice (1 min each), subsequent fixation in chilled 10% formalin buffer and storage at 4°C, protected from light [35].

To analysis of infarct injury, sections from all experimental groups were scanned and digitized within 7 post-fixation days of each experiment. The caudal surfaces of five contiguous sections were scanned and digitized using a scanner (HP Scanjet, HP, Omaha, NE, USA) and infarct area analyzed by Scion Image Software Application 4.0.3.2 (Scion, Frederick, MD. USA). Image regions from infarcted and non-damaged regions of the ipsilateral hemisphere and contralateral (non-ischemic, control) hemisphere were traced, calibrated and measured for area in mm^2^. The contralateral hemisphere (uninterrupted blood flow, no ischemia) was used as a control reference of area measurement for the ipsilateral side, normally supplied by right common carotid (permanently ligated, ischemic). Measurement of Infarct volume and total brain hemispheric volume expressed as (mm^3^) were calculated by adding the sum of all areas of five 2 mm thick coronal sections multiplied by thickness. To account for post-ischemic edema total right ipsilateral hemispheric infarct volume was calculated as the difference between total uninjured contralateral hemispheric volume and uninfarcted ipsilateral tissue volume [36] normalizing for the edema or atrophy associated with the ipsilateral side observed at the 24 h recovery time point. In addition, to control for possible changes in total brain hemisphere relative infarct volumes were also determined. To accomplish this ipsilateral infarct volumes were compared to total brain hemispheric volume, calculated as ipsilateral (right) side divided by twice the contralateral side ×100, and data expressed as infarct volume/total brain hemispheric volume %.

### Statistical Analysis

Statistical calculations were performed using the GraphPad Prism V. 4.01 software. The mean ± SD or SE were calculated for all samples, and significance was determined by either the Student's *t*-test or ANOVA with the Newman-Keuls or Bonferroni post hoc test. A value of *P*<0.05 was considered significant.

## Results

### OGD-mediated activation of CaMKII is associated with neural cell death in rat hippocampal slice cultures

**Figure 1:**
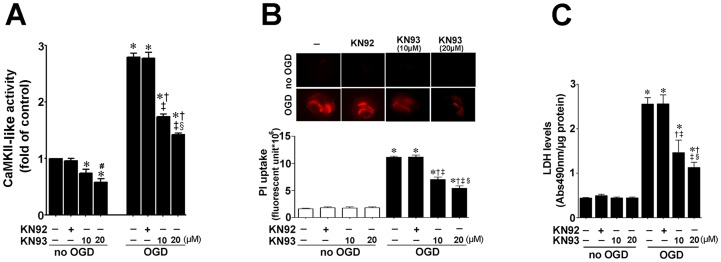
Oxygen glucose deprivation increases CaMKII activity which is correlated with cell death in rat hippocampal slice cultures. Rat hippocampal slice cultures were exposed to the CaMKII inhibitor, KN93 (10- or 20 µM) or its inactive analog, KN92 (10 µM) 2 h prior to OGD. Slices were harvested 2 h after OGD to determine effects on CaMKII activity. OGD increases CaMKII-like activity (A). The increase in CaMKII-like activity is dose-dependently attenuated by KN93, but not by KN92 (A). In addition the effect of OGD on cell death (B) and LDH release (C) were evaluated at 8 h after OGD. The effect on cell injury was quantified by measuring mean changes fluorescence due to PI uptake in the whole slice (B). KN93 attenuated both PI uptake and LDH release (B & C). The LDH absorbance at 490 nm was normalized by protein content. Data are presented as mean ± S.E from 4–8 independent experiments using 24 pooled slices per experiment. * *P*<0.05 vs. control, † *P*<0.05 vs. OGD alone, ‡P<0.05 vs. OGD+KN-92, § P<0.05 vs. OGD+10 µM KN93; #P<0.05 vs. no OGD+KN93 10 µM.

**Figure 2:**
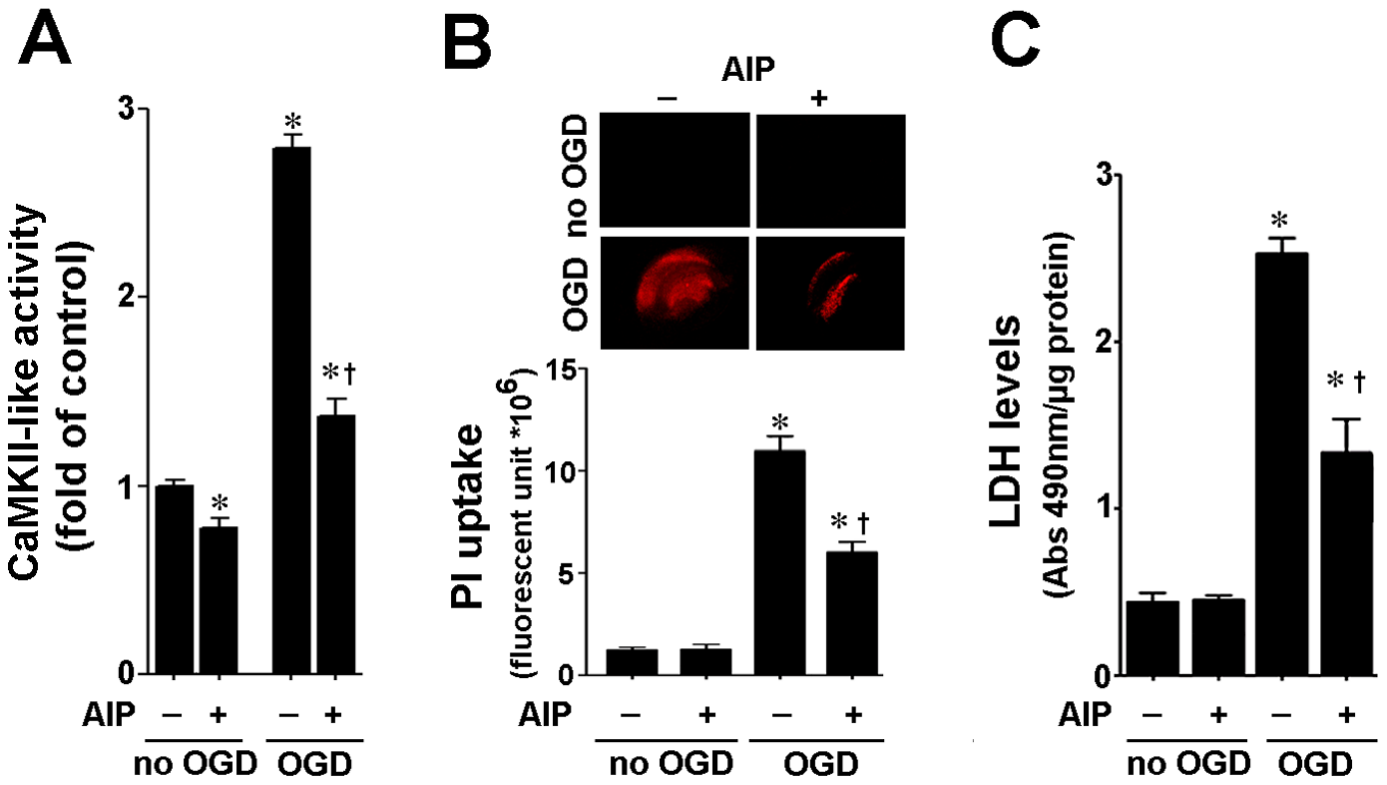
Autocamitide 2-related inhibitor peptide reduces cell death in rat hippocampal slice cultures expose to OGD. Rat hippocampal slice cultures were exposed to the CaMKII inhibitor, AIP (5 µM), 2 h prior to OGD. AIP significantly attenuated the OGD-mediated increase in CaMKII-like activity (A). AIP also significantly attenuated the increase in cell death (B) and LDH release (C) associated with OGD. Data are presented as mean ± S.E from 4 independent experiments using 24 pooled slices per experiment. * *P*<0.05 vs. control, † *P*<0.05 vs. OGD alone.

Hippocampal slices from P7 rats were pretreated with the CaMKII inhibitor, KN93 (10- or 20-µM, 2 h), or its inactive analogy, KN92 (10 µM, 2 h), then exposed to OGD. Two hours later we determined the effect of OGD on CaMKII activity. This time point was chosen as we have previously shown that the stress response kinase, p38MAPK is activated at this early time-point [16], [17]. Our data indicate that CaMKII activity is increased after OGD 2 h and that both doses of KN93, but not KN92, significantly inhibits this increase (Fig. 1 A). To examine the effect of CaMKII inhibition on OGD associated cell death, we quantified PI uptake in whole hippocampal slice 8 h after OGD. Again 8 h was chosen based on our previous studies demonstrating that OGD induced significant neural cell death at this time point [16], [17]. Our data demonstrate that OGD induces an increase in PI uptake (Fig. 1 B), KN93 significantly decreased cell death (Fig. 1 B). Similarly, KN93 significantly decreased lactate dehydrogenase (LDH) release (a measure of necrotic cell death) into the culture medium (Fig. 1 C). As pharmacologic agents have the potential for off-target effects, another potent CaMKII inhibitor, AIP was also utilized. AIP was effective in reducing CaMKII activity (Fig. 2 A) and the cell death after OGD (Fig. 2 B & C)

### CaMKII inhibition attenuates neural cell apoptosis in rat hippocampal slice cultures exposed to OGD

**Figure 3:**
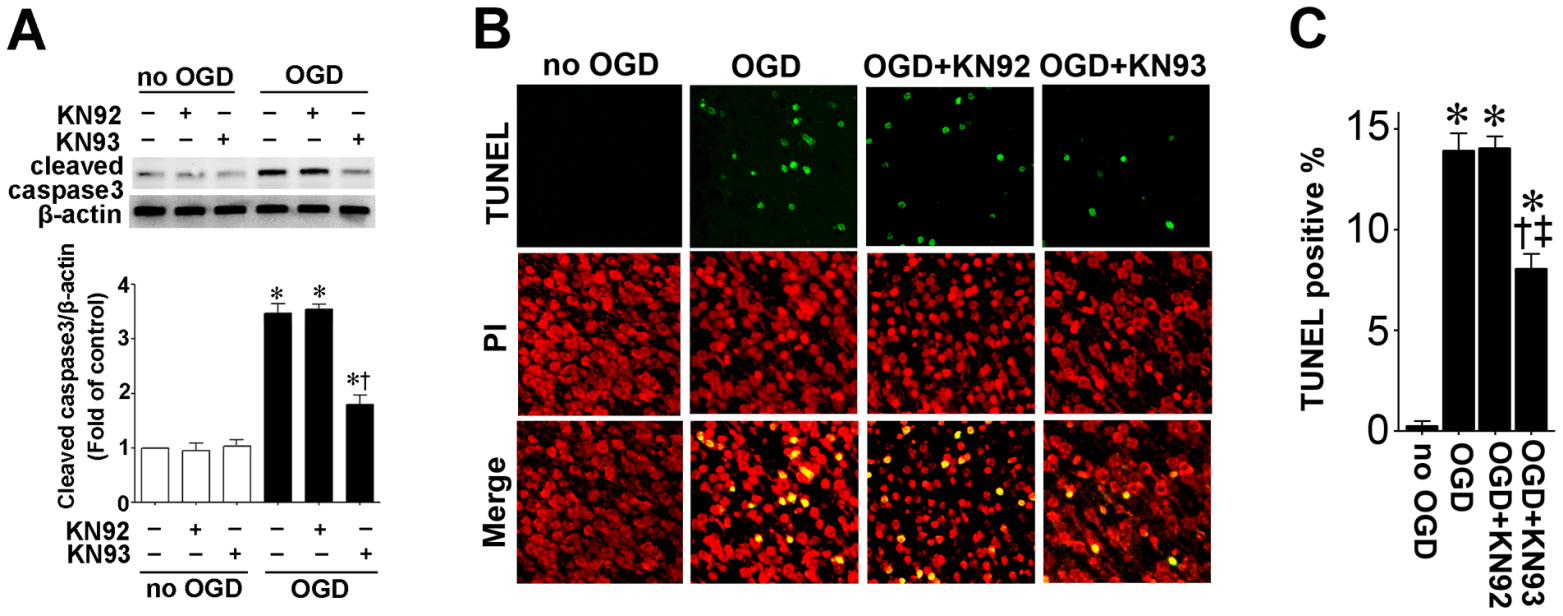
CaMKII inhibition attenuates apoptosis in rat hippocampal slice cultures exposed to oxygen glucose deprivation. Rat hippocampal slice cultures were exposed to OGD in the presence of the CaMKII inhibitor, KN93 or its inactive analog, KN92 (10 µM, 2 h prior to OGD). Slices were harvested 8 h after OGD and subjected to Western blot analysis to determine effects on cleaved caspase-3 (A). A representative blot is shown (A). OGD increases cleaved caspase-3 levels and this is attenuated by KN93, but not by KN92 (A). Slices were also subjected to TUNEL analysis. Representative images are shown demonstrating TUNEL staining of apoptotic cells (green) co-localized with PI staining of all the nuclei (red) (B). The magnification used was 10×. Quantification of the percentage of apoptotic nuclei to total nuclei was also carried out indicating that KN-93 pretreatment decreased the level of apoptotic nuclei in response to OGD (C). Data are presented as mean ± S.E from 4 independent experiments using 24 pooled slices per experiment. * *P*<0.05 vs. no OGD, † *P*<0.05 vs. OGD alone, ‡P<0.05 vs. OGD+KN92.

We next determined if CaMKII mediates neural cell death through the induction of apoptosis in neonatal slice cultures 8 h after OGD. Again 8 h was chosen based on our previous studies demonstrating that OGD induced significant neural apoptotic cell death at this time point [16], [17]. Using Western blot analysis, we found that cleaved caspase-3 level in the KN93 pretreated slice culture was significantly less than in that in the hippocampus slice cultures exposed to OGD and KN92 (Fig. 3 A). TUNEL staining also showed that KN93 significantly reduced OGD induced neural cell apoptosis (Fig. 3 B & C).

### OGD induced p38MAPK activation and p47^phox^ translocation are attenuated by CaMKII inhibition

**Figure 4:**
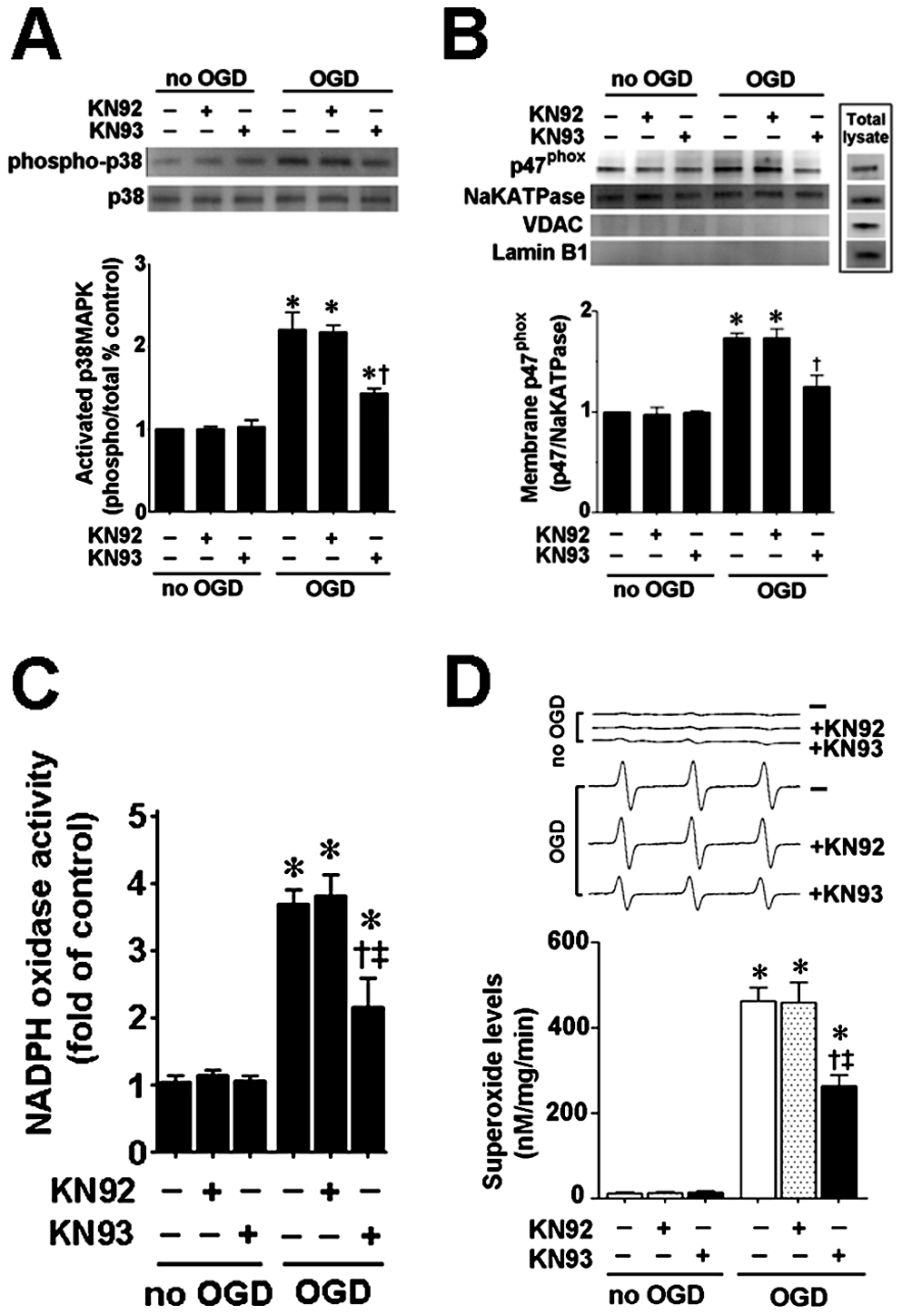
CaMKII inhibition attenuates p38MAP activation and p47^phox^ membrane translocation in rat hippocampal slice cultures exposed to oxygen glucose deprivation. Rat hippocampal slice cultures were exposed to OGD in the presence of the CaMKII inhibitor, KN93 or its inactive analog, KN92 (10 µM, 2 h prior to OGD), harvested 2 h after OGD and subjected to Western blotting using antibodies specific to phospho- and total-p38MAPK to estimate the effect on p38MAPK activation (A). OGD increases phospho-p38MAPK to total p38MAPK ratio and this is decreased by KN93 (A). OGD increases both the plasma membrane translocation of p47^phox^ (B) and NADPH oxidase activity (C) 2 h after OGD. KN93 pretreatment reduces both the OGD-mediated increase in p47^phox^ membrane translocation (B) and the increase in NADPH oxidase activity (C). Slices harvested 2 h after OGD were also subjected to electron paramagnetic resonance (EPR) using the spin-trap compound 1-hydroxy-3-methoxycarbonyl-2,2,5,5-tetramethylpyrrolidine HCl (CMH) to determine superoxide levels. Representative EPR waveforms are shown (D). Absolute levels of superoxide generation were determined as nmols superoxide generated/min/mg protein. KN93 pretreatment reduces the OGD-mediated increases in superoxide levels (D). Values are presented as mean ± S.E from 4 independent experiments using 24 pooled slices per experiment. * *P*<0.05 vs. no OGD, † *P*<0.05 vs. OGD alone, ‡P<0.05 vs. OGD+KN92.

We have recently shown that the activation of NADPH oxidase during neonatal brain HI is mediated by p38MAPK [16]. Thus, we next determined if CaMKII is upstream of p38MAPK activation. To accomplish this we used Western blot analysis to measure phospho-p38MAPK (to estimate p38MAPK activity) and membrane localized p47^phox^ protein levels after KN93 treatment. Two hours after OGD, phopsho-p38MAPK levels were significantly increased and this was attenuated by KN93 (Fig. 4 A). The attenuation of phospho-p38MAPK also correlated with a reduction in p47^phox^ membrane translocation (Fig. 4 B). In addition the increase in NADPH oxidase activity associated with OGD was attenuated by KN93 (Fig. 4 C) 2 h after OGD. EPR spectroscopy and spin trapping also demonstrated that the increase in superoxide generation associated with OGD was also correspondingly reduced (Fig. 4 D). Two hours was chosen for these studies based on our previous studies demonstrating that OGD induced significant activation of p38MAPK and NADPH oxidase at this time point [16], [17].

### CaMKII activity is increased in the neonatal HI rat brain and infarct volume is reduced by KN93

**Figure 5:**
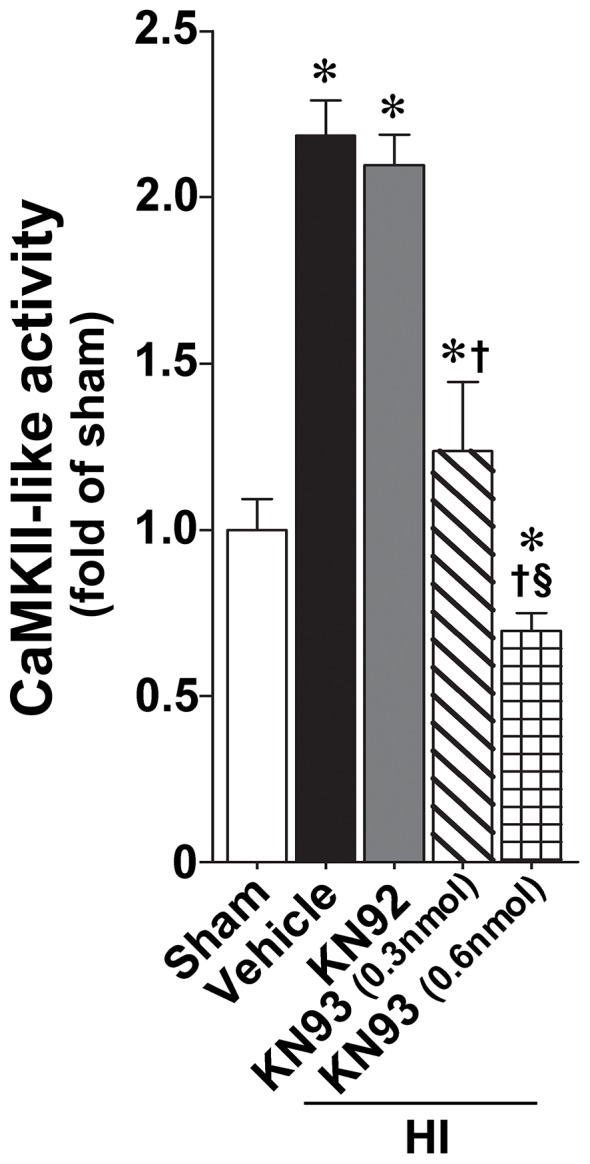
KN93 treatment attenuates CaMKII activity in the neonatal rat brain exposed to hypoxia-ischemia. P7 neonatal rats were pre-treated with KN93 (0.3 nmol and 0.6 nmol), the inactive analog KN92 (0.3 nmol), or vehicle then exposed to HI. Two hours after HI, CaMKII-like activity was determined in the right hemisphere of the brain and compared to the contralateral side. There is a significant increase in the right hemisphere that is dose-dependently attenuated by KN93, but not KN-92. Values are presented as mean ± S.E from 6 animals per group. *p<0.05 vs. sham, †p<0.05 vs. HI+vehicle, ‡P<0.05 vs. HI+KN92, § P<0.05 vs. HI+0.6 nmol KN93.

**Figure 6:**
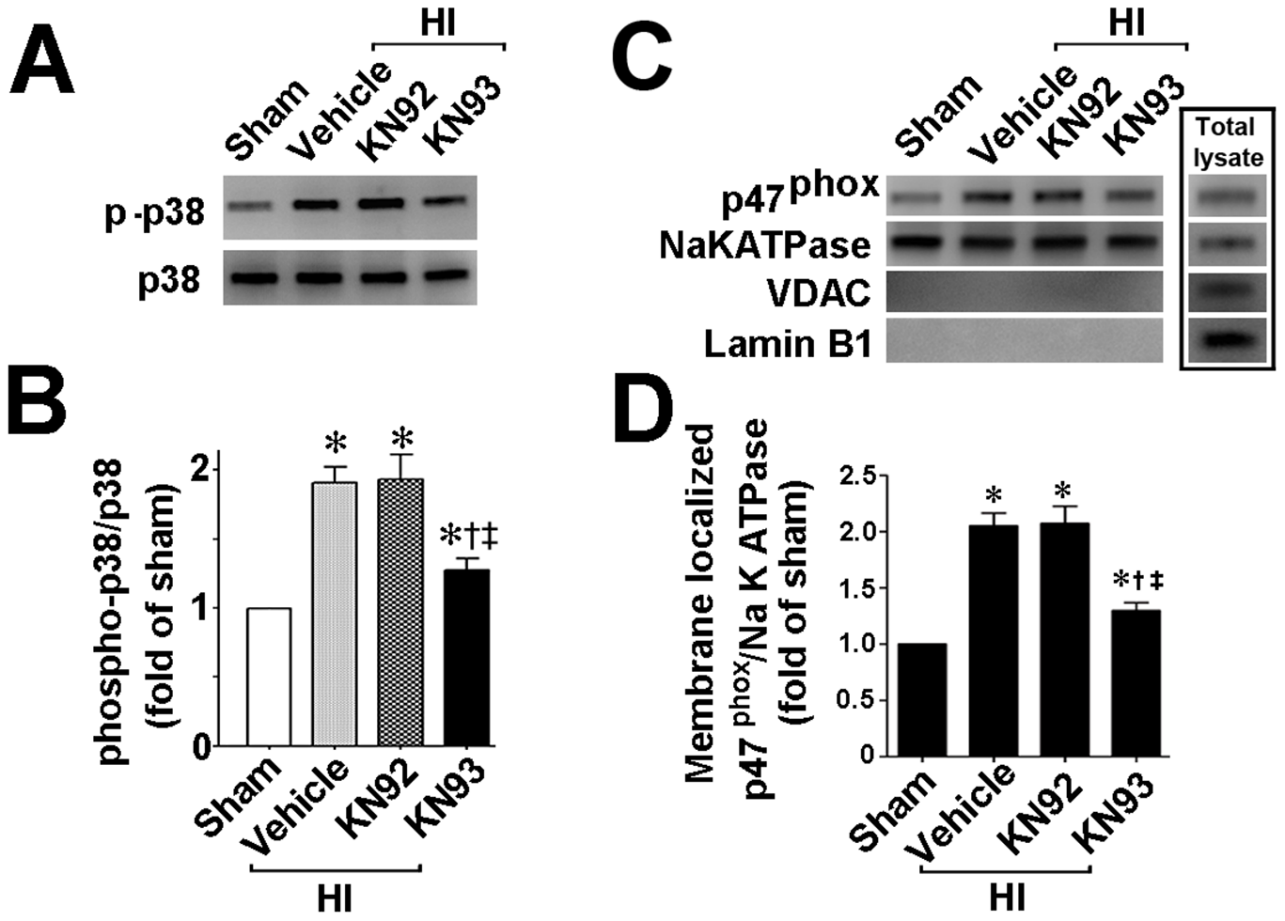
KN93 attenuates p38MAP kinase activation and p47^phox^ membrane translation in the neonatal rat brain exposed to hypoxia-ischemia. P7 neonatal rats were pre-treated with KN93 (0.3 nmol), the inactive analog KN92 (0.3 nmol), or vehicle then exposed to HI. Two hours after HI the brain was removed and the right hemisphere subjected to Western blotting to determine the effect on p38MAPK activation (estimated by ratio of phospho-p38MAPK to total p38MAPK) and p47^phox^ membrane translocation. KN93 pretreatment attenuates both the increase in phospho-p38MAPK (A) and the membrane translocation of p47^phox^ (B). Values are presented as mean ± S.E from 6 animals per group. *p<0.05 vs. sham, †p<0.05 vs. HI + vehicle, ‡P<0.05 vs. HI+KN92.

**Figure 7:**
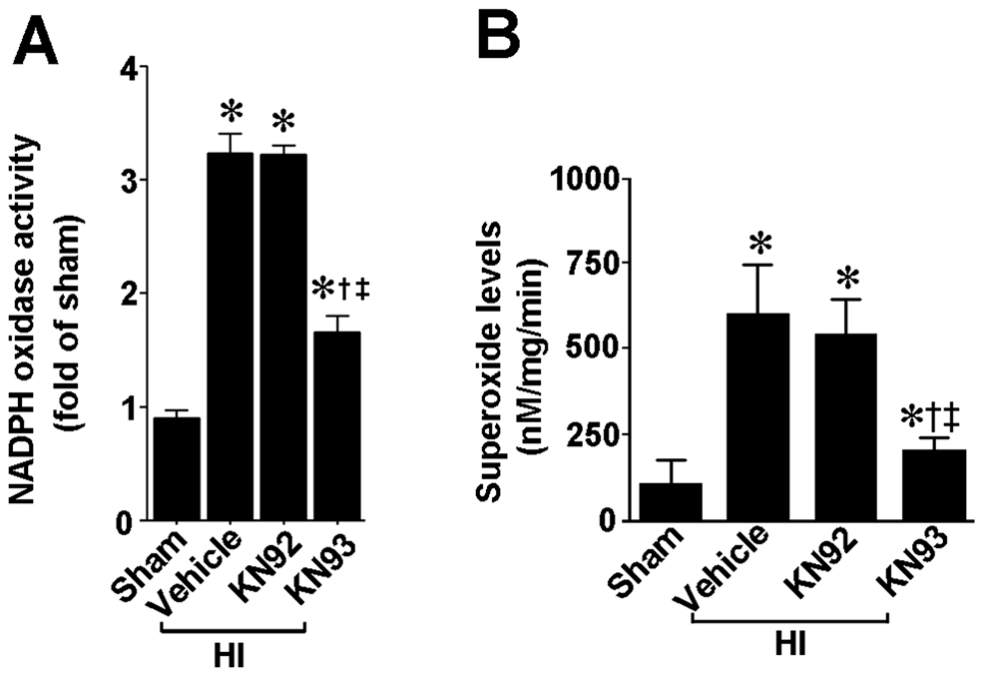
KN93 attenuates NADPH oxidase activity and superoxide generation in the neonatal rat brain exposed to hypoxia-ischemia. P7 neonatal rats were pre-treated with KN93 (0.3 nmol), the inactive analog KN92 (0.3 nmol), or vehicle then exposed to HI. Two hours after HI, NADPH oxidase activity (A) and superoxide levels (B) were determined in the right hemisphere of the brain. There is a significant increase in both NADPH oxidase activity and superoxide levels in the right hemisphere of the neonatal brain that is attenuated by KN93, but not by KN92. Values are presented as mean ± S.E from 6 animals per group. *p<0.05 vs. sham, †p<0.05 vs. HI+vehicle, ‡P<0.05 vs. HI+KN92.

We next used a neonatal rat model of HI to determine if CaMKII also regulates p38MAPK in vivo. Our data demonstrate that, two hours after HI, CaMKII activity is increased in the right hemisphere of HI brain compared to the contralateral side, used as a control. Further, the increase in CaMKII activity was attenuated by pretreatment with KN93 (0.3 nmol), but not by KN92 (0.3 nmol) (Fig. 5). The p38MAPK activation (Fig. 6 A & B) and p47^phox^ membrane translocation (Fig. 6 C & D) were also both reduced in the presence of KN93 (0.3 nmol). Further experiments also demonstrated that the increase in NADPH oxidase activity (Fig. 7 A) and superoxide levels (Fig. 7 B) induced by HI were attenuated by KN93 (0.3 nmol). Two hours was chosen for these studies as we have previously shown that there is already an increase in NADPH oxidase-derived superoxide in the right hemisphere of the rat brain at this time point [16], [17].

### KN93 reduces apoptotic death and infarct volume in a rat model of neonatal HI

**Figure 8:**
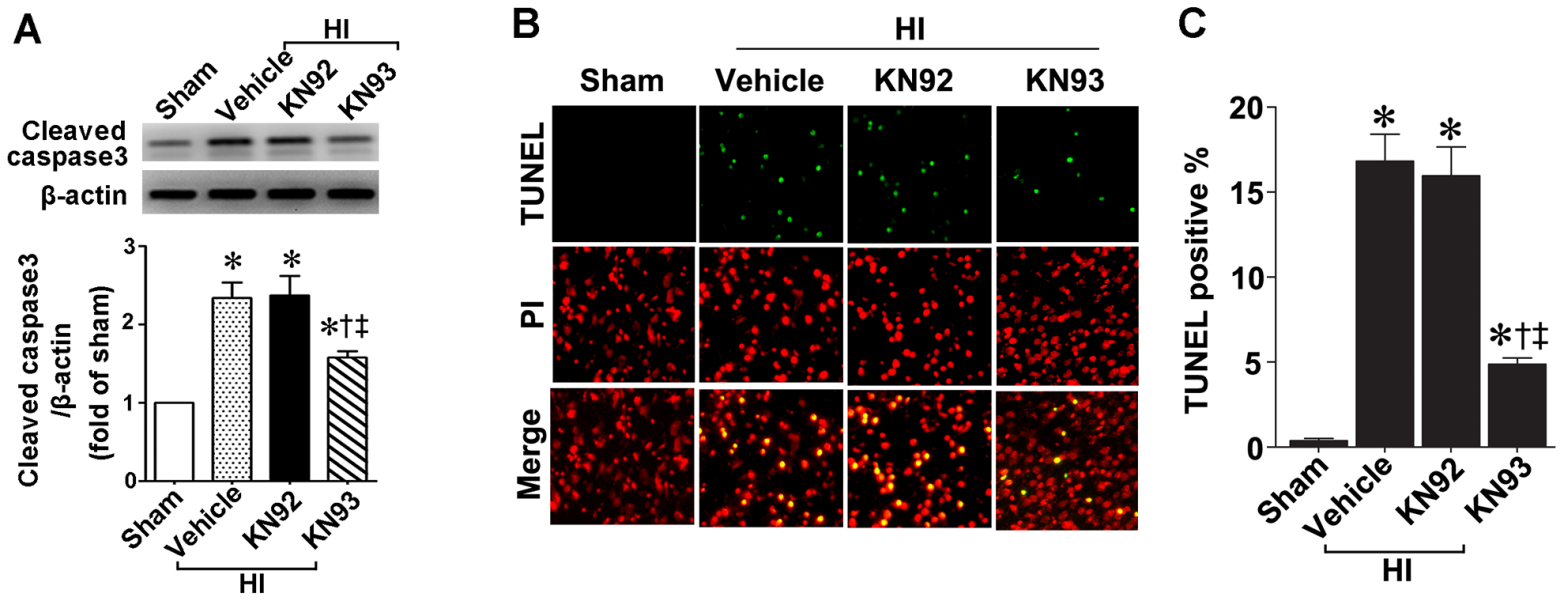
KN93 attenuates neural cell death in the neonatal rat brain exposed to hypoxia-ischemia. P7 neonatal rats were pre-treated with KN93 (0.3 nmol), the inactive analog KN92 (0.3 nmol), or vehicle then exposed to HI. Twenty-four hours after HI the brains were removed, the right hemisphere was subject to Western blot analysis to determine effects on cleaved caspase-3. A representative blot is shown (A). HI increases cleaved caspase-3 levels and this is attenuated by KN93, but not by KN92 (A). The right hemisphere was also sectioned and subjected to TUNEL staining to determine the effect on apoptosis in the neonatal brain. Sections were counterstained with PI (red) and representative images are shown (B). Quantitation of TUNEL positive cells shows that KN93, but not KN92, attenuates the increase in apoptosis in the right hemisphere by HI (C). Values are presented as mean ± S.E from 6 animals per group. *p<0.05 vs. left hemisphere, †p<0.05 vs. HI+vehicle, ‡P<0.05 vs. HI+KN92.

**Figure 9:**
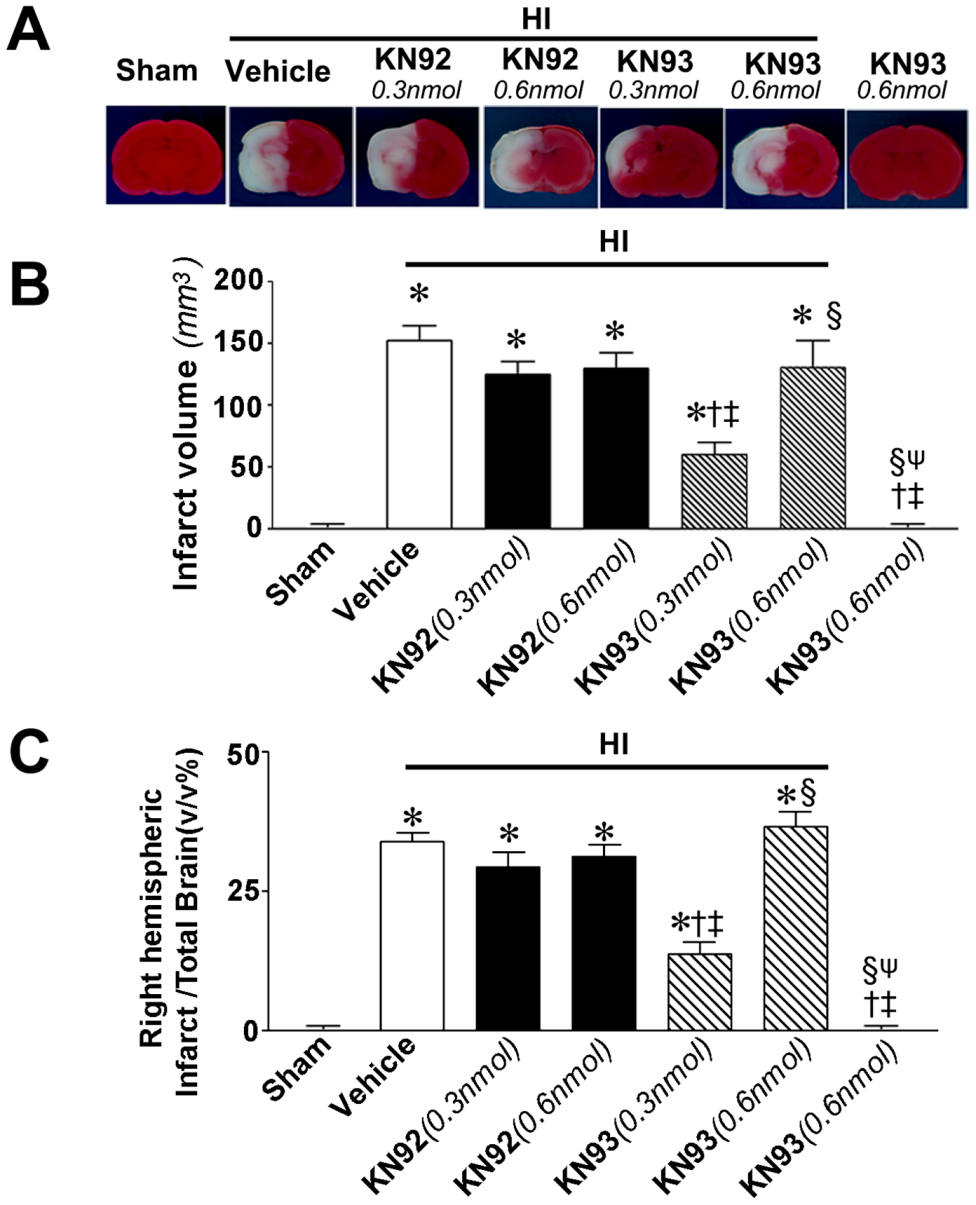
KN93 attenuates infract volume in the neonatal rat brain exposed to hypoxia-ischemia. P7 neonatal rats were pre-treated with KN93 (0.3- or 0.6 nmol), the inactive analog KN92 (0.3- or 0.6 nmol), or vehicle then exposed to HI. Twenty-four hours after HI the brains were removed, sectioned and subjected to TTC staining to determine changes in the infarct volume in the right hemisphere. Representative TTC stained sections are shown (A). KN93 (0.3 nmol), but not KN93 (0.6 nmol) or KN92 (either dose), reduces both the absolute infract volume (B) and relative infarct volume (C) associated with HI. Values are presented as mean ± S.E from 6 animals per group. *P<0.05 vs. left hemisphere, †p<0.05 vs. HI+vehicle, ‡P<0.05 vs. HI+0.3 nmol KN92, § P<0.05 vs. HI+0.3 nmol KN93, ψ P<0.05 vs. HI+0.6 nmol KN93.

Our data indicate that the increase in cleaved caspase 3 levels (Fig. 8 A) and TUNEL positive nuclei staining (Fig. 8 B & C) in the right hemisphere of the brain 24 h after neonatal HI are both reduced by pretreatment with KN93. The infarct volume 24 h post-HI was also quantified. Our data indicate that KN93 at 0.3 nmol significantly reduced both the absolute infract volume (Fig. 9 B) and the relative infarct volume (Fig. 9 C) in the right hemisphere. However, neither KN93 at 0.6 nmol nor KN92 (0.3 nmol) produced any reduction in infract volume (Fig. 9) despite 0.6 nmol KN93 inhibiting CaMKII activity (Fig. 5). Twenty-four hours was chosen as our previous studies have shown that at this time point there is significant apoptotic cell death and a significant increase in infarct volume [16], [17].

## Discussion

It is generally recognized that oxidative stress and Ca^2+^ toxicity are the main mechanisms of HI brain injury. CaMKII although found in most of the tissues is predominant in the brain where it makes up to 1–2% of the total protein in the cerebral cortex and the hippocampus [37]. It is enriched at the post-synaptic density (PSD), a localization which is thought to be critical for many of its proposed neural functions. In particular, this kinase is widely believed to be a critical mediator of long term potentiation (LTP), which underlies learning and memory processing [38]. CaMKII is activated when increases in intracellular Ca^2+^ leads to the binding of Ca^2+^/calmodulin complexes [39]. This study adds to this body of literature by suggesting that the brain injury associated with neonatal HI involves, at least in part, NADPH oxidase generated ROS that appears to be mediated by the activation of CaMKII and p38MAPK.

Increases in CaMKII activity have been reported to correlate with cell death in several different animal models. For example, it has been shown that CaMKII is activated during ischemia/reperfusion (IR) injury in the heart [40] and CaMKII activation has been shown to correlate with increases in cardiac caspase-3 activity [40]. Further, during IR injury in the heart, CaMKII inhibition, using KN93, diminished caspase-3 activity, LDH release, TUNEL positive nuclei and infarct size [41], [42]. Similarly, in a transient global ischemia rat model, it has been demonstrated that CaMKII contributes to ischemic neural death through the phosphorylation of acid-sensing ion channels (ASICs) that directly mediate neural cell death [43]. However, the role of CaMKII in HI mediated brain injury less unclear. In our neonatal HI model we observed increased CamKII activity and neural protection with CamKII inhibitors. However, studies in the mature rat brain have shown that translocation and inactivation of CaMKII activity parallels neural damage [44], [45], [46]. While another report correlated increased CaMKII expression with neural cell death [47]. The discrepancies between these data may be due to the differences with the HI model used, the intensity of the insult, or the time point of observation. Alternatively, as CaMKII is also developmentally regulated in the brain, differences in CaMKII expression between the neonatal and mature brain may impact the response to HI injury. The growth spurt in the brain of rats and mice occurs in the first 3–4 weeks of life, reaching its peak around postnatal day P10 [48]. In this period of dynamic brain development, there is a striking increase in CaMKII expression. In mice, the amount of CaMKII in the brain increases dramatically from P1 to P28, especially between P7 and P14, about 23-, 23- and 25 fold increase in cortex, hippocampus and whole brain respectively [49]. In the rat, CaMKII increases dramatically between P10 and P30 [50]. This developmental up-regulation of CaMKII is associated with synaptogenesis [51]. In mature neurons, the levels of CaMKII are both higher, and more stable, than the neonatal brain [52]. One recent study in a rat neonatal HI brain injury model, demonstrated that phospho-CaMKII-Thr286 is transiently elevated after HI [53]. It has been suggested that early transient phosphorylation or activation of CaMKII may sensitize glutamate receptors and contribute to the vulnerability of CaMKII-expressing neurons after HI, leading to the interruption of synapse development, synaptic remodeling and plasticity [4], [6]. Consistent with these results, our data both in hippocampal slice cultures and the neontal rat brain, demonstrate that CaMKII activity is elevated early after HI and this correlates with neural cell death at later time points. The complexity of the role of CaMKII in neural health is also shown by our data in which we observed less CaMKII activity with a higher concentration of KN93, in both hippocampal slice cultures and the neonatal rat brain exposed to HI. However, we only observed increased neuroprotection in slice cultures. In fact at the highest dose of KN93 used in vivo, 0.6 nmols, we found no evidence of a reduction in infract volume. At first glance this is a puzzling outcome. However, it has been reported that a sustained loss of CaMKII enhances the sensitivity of neurons to glutamate-mediated toxicity [54], [55]. Further, the genetic knockout of CaMKIIα also increased the infarct size in a mouse model of stroke [56]. The mechanism of this shift of CaMKII inhibition from neuroprotection to enhanced neural vulnerability is not clear. However, it suggests that some basal activity of CaMKII is required to maintain a neural cell in a viable state. It is also worth noting that the syntide-2 peptide substrate we used to measure CamKII activity can also be a substrate for other Ca^2+^-dependent kinases including protein kinase C, phosphorylase kinase and myosin light chain kinase [28]. Thus, although CaMKII has the highest rate of catalysis and strongest affinity for syntide-2 we may be either under- or over-estimating the effect of OGD on CamKII activity.

Our data also indicate that the mechanism by which KN93 is neuroprotective is, at least in part, through a reduction in ROS generation. The neonatal brain is highly susceptible to ROS [57]. ROS play an important role in neonatal HI brain injury. We have recently shown the important role played by NADPH oxidase derived ROS in the brain injury associated with neonatal HI [16], [17]. Using the NADPH oxidase inhibitor, apocynin or a siRNA approach to reduce p47^phox^ translocation to the membrane we were able to significantly reduce the increase in superoxide associated with OGD in hippocampal slice cultures. Superoxide levels were also attenuated in neonatal rat model of HI treated with a gp^91phox^ docking sequence peptide to reduce the interaction of gp91^phox^ with p47^phox^. The reductions in superoxide led to increased neural survival [16], [17]. Further, our data suggest that CaMKII indirectly activates NADPH oxidase activity via p38MAPK. However, it is possible that there may also be direct effects on the NADPH oxidase complex itself.

In this study we have also investigated the interactions between CaMKII and p38MAPK in HI brain. We found that in hippocampal slice cultures exposed to OGD or the neonatal rat brain exposed to HI, the activation of p38MAPK is attenuated by CaMKII inhibition. Recent reports have shown that the expression and phosphorylation of MAPKs is altered in the post-ischemic brain, and that inhibition of MAPK cascades can alter the outcome of ischemic brain injury in animal models [58], [59], [60], [61]. Indeed our previous studies have shown that OGD leads to a transient increase in p38MAPK activation in rat hippocampal slice cultures and that this, in turn, leads to increased superoxide generation and neural cell death [17]. Other studies in animal models have also shown that the expression of p38MAPK changes in the ischemic brain and that inhibition of p38MAPK improve the outcome of ischemic brain injury [62], [63]. Our data indicate that CaMKII inhibition attenuates the p38MAPK dependent increase in p47^phox^ membrane translocation and reduces the generation of superoxide from NADPH oxidase. Together these data strongly suggest that CaMKII and p38MAPK are functionally coupled to NADPH oxidase and both are required to produce neural death during HI. Our data also adds more support to the idea that there is crosstalk between calcium and ROS signaling in HI. The MAPK pathway is typically composed of a highly conserved MAPK module including three kinases, namely MAPK kinase kinase (MKKK), MAPK kinase (MKK), and MAPK. It is suggested that upstream MAPK kinase kinase, can activate MAPK kinase, which subsequently phosphorylate p38MAPK at Thr180 and Tyr182 [64], [65], [66], [67]. From our studies we are not able to determine if CaMKII acts as a MKKK or a MKK, i.e., is directly phosphorylating p38MAPK or acts further upstream. Indeed it has been shown that CaMKII can directly phosphorylate the MKKKs, TAK1 [68] and ASK1 [69], which lead to activation of JNK and p38MAPK, suggesting that other as yet undescribed kinases may also be involved in this process. Further, there may also be CaMKII-independent activation of p38MAPK during HI. Previous studies have shown that p38MAPK can also be activated by nitric oxide (NO) [70], [71], [72]. As HI results in an increase in NO release from neural NO synthase [73], [74], [75], [76], [77], [78], [79], NO-mediated p38MAPK activation may also be involved in the activation of NADPH oxidase. However, further studies will be required to test this possibility.

In conclusion, our results, both from hippocampal slice culture expose to OGD and the neonatal rat exposed to HI, demonstrate that CaMKII activation is linked to p38MAPK signaling and NADPH oxidase generated superoxide. These findings suggest that CaMKII plays an important role in the cross-talk between Ca^2+^ and ROS signaling during the development of the neonatal brain injury associated with HI and may be a potential therapeutic target for neonatal HI, a pathologic process for which there is no effective treatment.
